# Study of child and adolescent forensic psychiatry : procedure and clinical profile Abstract

**DOI:** 10.1192/j.eurpsy.2023.1212

**Published:** 2023-07-19

**Authors:** R. Gadhoum, M. Hamza, H. Rezgui, H. Ben Youssef, A. Ben Hamouda, M. Daoued, S. Bourgou, F. Charfi, A. Belhadj

**Affiliations:** Child and Adolescents psychiatry, Mongi Slim Hospital, Tunis, Tunisia

## Abstract

**Introduction:**

Forensic psychiatry is a specific field of practice in child psychiatry where skills are applied for legal purposes and the expert is faced with certain difficulties.

**Objectives:**

Our objective was to determine the characteristics of the forensic procedure and the clinical profile of the consultants.

**Methods:**

Cross-sectional and retrospective descriptive study concerning patients consulting the child psychiatry department of Mongi Slim Hospital between January 2018 and December 2019 in the context of a forensic expertise, excluding administrative expertise. Telephone interviews were carried out for the medicolegal follow-up.

**Results:**

The average age was 9 [+/- 3.1] years and the sex ratio was 0.97. The majority of the requests for expertise came from the specialised unit for violence against women and children (40%). The expert’s task was to carry out a child psychiatric examination in 44% of cases and to establish credibility in 6.8% of cases. Apart from 3 cases of perpetrators, the requests concerned victims of physical (40.1%), sexual (38.6%) and psychological (21.3%) aggression. The assault had taken place within the family in 52% of cases and the alleged aggressor was the father in 36%. The average time between the incident and the request for an expert opinion was significantly longer in cases of sexual assault (p =0.01). The physical assaults concerned more boys (p = 0.05) while those of a sexual nature concerned female children (p=0.005). Victims of sexual aggression showed more sexualised behaviour (p<0.05). No significant differences were found in psychiatric diagnoses according to the type of assault, but an examination without abnormalities was prevalent in cases of psychological assault (p=0.009). Claims were significantly more difficult to interpret in preschool children (p=0.05). Child victims of sexual abuse had more subsequent requests for further investigations (p=0.05) and a judgment, pronounced in 34% of cases, was most often related to psychological abuse (p=0.05).

**Conclusions:**

The practice of forensics requires a specific competence that requires extensive training. Better coordination between the professionals involved is needed as well as the creation of specialised forensic units.

**Disclosure of Interest:**

None Declared

